# Editorial: Plant-Based Products, Phytochemicals and Glycemic Control

**DOI:** 10.3389/fendo.2022.906690

**Published:** 2022-04-28

**Authors:** Alessandro Leone, Gelsomina Fico, Simona Bertoli, Alberto Battezzati

**Affiliations:** ^1^International Center for the Assessment of Nutritional Status (ICANS), Department of Food, Environmental and Nutritional Sciences (DeFENS), University of Milan, Milan, Italy; ^2^Department of Pharmaceutical Sciences (DISFARM), University of Milan, Milan, Italy; ^3^Botanic Garden G.E. Ghirardi, Department of Pharmaceutical Sciences (DISFARM), University of Milan, Toscolano Maderno, Italy; ^4^Lab of Nutrition and Obesity Research, Istituto Auxologico Italiano, Istituti di Ricovero e Cura a Carattere Scientifico (IRCCS), Milan, Italy

**Keywords:** medicinal plants, phytochemicals, phenolics, obesity, diabetes, glucose, insulin, metabolic health

Diabesity is a major public health problem that has reached epidemic proportions globally. The rapid increment of obesity and type 2 diabetes (T2D) prevalence is closely associated with economic development and the “nutrition transition”, that is, changes in dietary habits that have occurred in recent decades, associated with changes in the way food is produced, distributed, and consumed (1). In recent decades, we have witnessed a process of diet westernization and the introduction of ultra-processed foods that are energy dense and of low nutritional quality. These foods attract the attention of consumers because they are hyperpalatable, ready-to-eat, packaged in attractive packaging, and aggressively advertised. Nevertheless, several epidemiological studies reported their consumption associated with the risk of obesity and diabetic syndrome (2–5). The nutrition transition occurred first in high-income countries, but is now rapidly occurring in low- and middle-income countries. This is exposing all populations, at varying rates, to multiple factors that affect metabolism and cardiometabolic physiology (6).

Lifestyle modifications aimed to improve diet nutritional quality and increase physical activity represent the first line of intervention for the treatment of obesity and T2D, but implementation remains difficult (7). For this reason, there is increasing recourse to the second line of intervention, which involves the use of oral hypoglycemic drugs. At present, there are many antidiabetic drugs available to treat hyperglycemia, that specifically act through improving insulin sensitivity, supplementing insulin, increasing insulin secretion and stimulating glucose uptake. However, some antidiabetic drugs are compromised with several undesirable side effects such as gastrointestinal disorders, liver failure, weight gain, tachycardia, and hypothyroidism. In addition, the increased use of hypoglycemic drugs, especially newer generation drugs, leads to increased health care expenditures, which are often unsustainable for developing countries where there are barriers to accessing health care and essential medicines, and where there is a lack of funding and reliable supply systems (8).

The food environment represents a resource of relatively safer phytochemicals and bioactive compounds that can improve glycemic control and promote health status. In their review, Sarkar et al., examined the role of plant food sources of phenolic bioactive metabolites for countering chronic oxidative stress, chronic hyperglycemia and improving digestive health, which are considered key targets on which to act to prevent the risk of T2D and its comorbidities. The authors also highlight how phenolic metabolite content and health-protective functional qualities are influenced by plant genotypes, differences in growing environment, agricultural and horticultural production practices, and postharvest factors. It follows that screening and optimization of the different factors in the food chain that have a significant impact on the functional quality related to the phenolic bioactives of plant foods, are essential steps to design targeted health foods with optimal phenolic levels. Marton et al. systematically reviewed the randomized control studies testing the effect of *Curcuma longa* or curcumin on metabolic status of diabetic patients. The authors analyzed 16 studies and found that the administration of *C. longa* or curcumin in a dose ranged from 80 mg to 2100 mg per day for a period of 8-16 weeks significantly reduced lipid peroxidation, fasting glucose, glycated hemoglobin, triglycerides, total and LDL cholesterol, C-reactive protein and blood pressure. Moreover, the treatment increased HDL cholesterol and serum antioxidant capacity. It follows that the use of *C. longa* or curcumin can be considered in the therapeutic approach in T2D patients to improve glycemic control and prevent the development of micro- and macro-vascular complications. Medicinal plants, as well as plant foods, are also sources of phytochemicals and bioactive compounds with antidiabetic potential. For centuries, humans have used plants as a remedy for numerous ailments, including diabetes and its comorbidities, and some modern antidiabetic pharmaceuticals were originally isolated from plants or derived from phytochemicals. One example is metformin, a first-line hypoglycemic drug widely used to treat T2D, derived from the extract of *Galegine officinalis*. Numerous plants, however, have shown hypoglycemic potential at different levels of evidence. In their review, Alam et al. reviewed the most notable medicinal and dietary plants along with their isolated antidiabetic phytochemicals to give new insights into the development of novel functional foods and drugs against diabetes. The authors also describe the mechanisms by which bioactive compounds are thought to act to improve glycemic control. Specifically, they would improve T2D through six mechanisms of action, including increased insulin secretion and sensitivity, glucose uptake by muscle cells and adipose tissue and inhibition of glucose absorption from the intestine and glucose production by hepatocytes along with demonstrating anti-inflammatory properties. Moreover, some phytochemicals from medicinal and dietary plants may regulate epigenome in chronic administration. In their review, Ramirez-Alarcon et al. discuss of phytochemical that regulate the activities of common protein targets for treatment of T2D. The authors also discuss the mechanisms involved in the therapeutic effects of potential epidrugs, such as decreasing insulin resistance through inhibition of 11β-HSDs, enhancing insulin activity through inhibition of PTP1B, regulation of estradiol through inhibition of 17β-HSDs, regulation of glucose input into hexosamine biosynthesis through inhibition of GFAT, and the role of SIRT6 in insulin secretion, glucose uptake, and regulation of gluconeogenesis. Finally, in their review, Boudreau et al. focused on the effects of *Artemisia scoparia* on metabolic health. Particularly, the authors examined *in vitro* and *in vivo* studies reporting the ability of *A. scoparia* and its bioactive constituents in mitigating the obesity- and diabetes-related metabolic dysfunctions. As main findings, they report the plant extract enhancing adipocytes differentiation and lowering fasting insulin and glucose levels in animal model, while inducing metabolically favorable changes in liver and adipose tissue.

In conclusion, some lines of evidence suggest that medicinal and dietary plants, along with isolated bioactive compounds, have the potential to improve glycemic control and metabolic health, preventing the development of T2D and its complications. However, further clinical studies are needed to support the currently available evidence. If confirmed, medicinal and dietary plants would represent an attractive, easily accessible, low-cost, and sustainable source of numerous bioactive compounds useful in the development of novel functional foods, nutraceuticals, and replacement/complementary therapies for traditional antidiabetic drugs.

## Author Contributions

AL: Writing - original draft; GF: Writing - review & editing; SB: Writing - review & editing; AB: Writing - review & editing. All authors contributed to the article and approved the submitted version.

## Conflict of Interest

The authors declare that the research was conducted in the absence of any commercial or financial relationships that could be construed as a potential conflict of interest.

## Publisher’s Note

All claims expressed in this article are solely those of the authors and do not necessarily represent those of their affiliated organizations, or those of the publisher, the editors and the reviewers. Any product that may be evaluated in this article, or claim that may be made by its manufacturer, is not guaranteed or endorsed by the publisher.
